# Contribution of BRCA1 5382insC mutation in triple negative breast cancer in Tunisia

**DOI:** 10.1186/s12967-019-1873-8

**Published:** 2019-04-11

**Authors:** Wijden Mahfoudh, Inchirah Bettaieb, Randa Ghedira, Kaouther Snoussi, Nadia Bouzid, Zahra Klayech, Sallouha Gabbouj, Yassmine Remadi, Elham Hassen, Noureddine Bouaouina, Abdelfateh Zakhama

**Affiliations:** 1 Laboratory of Molecular Immuno‑Oncology, Faculty of Medicine of Monastir, Monastir University, 5019 Monastir, Tunisia; 2 Higher Institute of Biotechnology of Monastir, Monastir University, Monastir, Tunisia; 3 Department of Cancerology and Radiotherapy, Farhat Hached University Hospital, Sousse University, Sousse, Tunisia

**Keywords:** Triple negative breast cancer (TNBC), *BRCA1*, 5382inC mutation, Tunisia

## Abstract

**Background:**

Triple negative breast cancer (TNBC) has been classified as a disease subgroup defined by the lack of expression of estrogen and progesterone receptors as well as the absence of the human epidermal growth factor receptor-2 (HER2) overexpression. Germline mutations in the *BRCA1* gene have been associated with TNBC. Approximately 70% of breast cancers arising in BRCA1 mutation carriers and up to 23% of breast cancers in BRCA2 carriers display a triple negative phenotype. However, the contribution and the frequency of BRCA1 mutations in individuals with TNBC, not specifically selected for age at diagnosis or enriched family history of breast/ovarian cancer, have not been investigated in the Tunisian population and are to be established. The aim of the present study was to assess the contribution and the prevalence of recurrent BRCA1 germline mutation (5382inC) in Tunisian women with TNBC unselected for family history or age at onset.

**Methods:**

For BRCA1 5382inC mutation detection, the exon 20 coding region and exon–intron boundaries of *BRCA1* was analyzed using direct DNA sequencing. A total of 33 DNA samples from Tunisian women diagnosed with TNBC and unselected for family history or age at onset were analyzed.

**Results:**

The 5382inC mutation was identified in 2 out of 33 women with TNBC with an overall prevalence of 6% (2/33). The detection rate of the 5382inC mutation among TNBC women with family history of breast cancer was 25% (2/8). The two 5382inC mutation carriers were postmenopausal and diagnosed at the age of 50 and 57. When stratified by age, the frequency of BRCA1 mutation in patients diagnosed at age ≥ 50 years was 8.7% (2/23).

**Conclusions:**

Our results confirm a noticeable contribution of BRCA1 5382inC mutation in TNBC development in Tunisia and further indicate that screening for 5382insC mutation in the *BRCA1* gene is of interest in genetic testing in our population. Additionally, our data highlight that receptor triple negativity could be an effective selection criterion for *BRCA1* genetic test in our population and should therefore be considered in genetic testing guidelines in Tunisia.

## Background

Breast cancer (BC) is the most prevalent cancer among women worldwide. Its incidence is on the rise all over the world, especially in developing countries [1]. In Tunisia, it represents the most common malignancy in women. In 2012, it accounted for 33.5% of all female cancers with 1826 new cases and an estimated incidence of 31.8 per 100,000 (IARC 2012). Compared to European and American populations, breast cancer in Tunisia occurs at a mean age of 10 years younger with high rate among women younger than 35 years (11%) [2].

Breast cancer is currently identified as a complex disease including a heterogeneous group of tumors. Triple negative breast cancer (TNBC) has been classified as a disease subgroup defined by the lack of expression of estrogen and progesterone receptors as well as the absence of the human epidermal growth factor receptor-2 (HER2) overexpression. TNBC accounts for 15 to 20% of breast cancer cases [3]. It is associated with aggressive behavior and a worse prognosis than other breast cancer subtypes [4]. Recently, a descriptive analysis of molecular subtypes in 966 Tunisian breast cancer cases has showed that the triple negative subtype has a higher incidence in Tunisia compared to Western countries (22.5%) and is significantly associated with large tumor size, younger age and high grade [5].

Germline mutations in the *BRCA1* gene are associated with TNBC. Approximately 70% of breast cancers arising in BRCA1 mutation carriers and up to 23% of breast cancers in BRCA2 carriers display a triple negative phenotype [6]. In Tunisia, it has been shown that BRCA1 mutation carriers have a higher incidence of triple negative subtype (75%) than BRCA2 mutation carriers (0%) [7]. However, the contribution and the frequency of BRCA1 mutations in individuals with TNBC, not specifically selected for age at diagnosis or enriched family history of breast/ovarian cancer, are not investigated in the Tunisian population and are to be established.

To date, only a few studies, including of our group, have investigated the spectrum and frequency of BRCA1 mutations in Tunisia [8–10]. These studies reported a limited number of BRCA1 mutations. A study showed that screening for common mutations in *BRCA1* allowed the detection of a substantial percentage of mutations in the Tunisian population. Therefore, such an approach may be of interest in genetic testing of high-risk women in Tunisia allowing a more rapid and less expensive test [11]. The cumulative results of the Tunisian studies indicated the predominance of two BRCA1 recurrent mutations: c.5266dupC (5382inC) and c.211dup (330insA). The 5382inC mutation was detected in all Tunisian series including ours. Therefore, the aim of the present study was to assess the contribution and the prevalence of the recurrent 5382inC BRCA1 germline mutation in Tunisian women with TNBC, regardless of family history or age of diagnosis.

## Methods

### Study population

The participants were recruited between 2008 and 2012 at the Department of Cancerology and Radiotherapy of Farhat Hached University Hospital of Sousse, Tunisia. During blood sampling, participants were interviewed using a questionnaire to collect demographic characteristics, personal and family history of cancer, menopausal status, reproductive behavior information and contraceptive methods. Breast cancer women were selected based on triple negative status and regardless of the age at onset and family history of cancer. A total of 33 women diagnosed with triple negative breast cancer were included in the present study. ER, PR and HER2 status were confirmed in a histopathology report of the tumor samples. A detailed description of the clinico-pathological characteristics of the study cohort is presented in Table 1.

**Table 1:** Characteristics of the study cohort

Characteristics	Patients (%)
Total	33
Family history	
Yes	9 (27.3)
No	24 (72.7)
Age at diagnosis	
< 50	10 (30.3)
≥ 50	23 (69.7)
Menopausal status	
Non menopausal	12 (36.4)
Menopausal	21 (63.6)
Tumor size	
T1–T2	19 (57.6)
T3–T4	14 (42.4)
SBR grade	
SBR 1–2	14 (42.4)
SBR 3	19 (57.6)
Lymph node status	
Negative	17 (51.5)
Positive	16 (48.5)
Metastasis	
Negative	27 (81.8)
Positive	6 (18.2)

The study was approved by the National Ethical Committee and a written informed consent was obtained from all enrolled patients prior to their participation.

### Genomic DNA extraction

Genomic DNA was extracted from peripheral blood leukocytes using the standard salting out procedure  [12]. Briefly, 10 ml of blood were mixed with Triton lysis buffer (0.32 M sucrose, 1% Triton X-100, 5 mM MgCl_2_, 10 mM Tris–HCl, pH 7.5). The pellet was incubated with proteinase K at 56 °C and subsequently salted out using a saturated NaCl solution. The precipitated proteins were removed by centrifugation. The DNA in supernatant fluid was precipitated with ethanol. Finally, the DNA pellet was conserved in Tris-EDTA buffer. DNA concentration and quality were analyzed by thermo-scientific NanoDrop 2000™.

### BRCA1 5382inC mutation detection

As for BRCA1 5382inC mutation detection, the exon 20 coding region and exon–intron boundaries of *BRCA1* was analyzed using direct DNA sequencing. Exon 20 was amplified in 20 μl with 100 ng DNA, 1× reaction buffer, 200 μM dNTPs, 1.5 mM MgCl_2_, 0.8 μM primers (designed by Centre Jean Perrin, sequences available on request) and 1 unit Taq polymerase (except primers, all other reagents from Promega, France). PCR was performed in an thermocycler (Biometra, Germany) with an initial denaturation at 94 °C for 5 min, followed by 30 cycles of (94 °C 20 s, 54 °C 20 s, 72 °C 20 s). After amplification, the PCR products were subjected to electrophoresis in a 2% agarose gel. The product was cut from the gel and purified using QIAquick gel extraction kit (Qiagen, CA). Both DNA strands were sequenced using BigDye Deoxy terminator cycle sequencing kit (BD V3.1, Applied Biosystems). Cycle sequencing consisted of an initial denaturation at 96 °C for 10 min followed by 25 cycles of 96 °C for 10 s, 50 °C for 5 s, 60 °C for 4 min. The product was purified on a separation column (AutoSeq™ G-50, Amersham Biosciences), and the templates were sequenced on an automated ABI-PRISM 310 Genetic Analyzer (Applied Biosystems). Sequence analysis was performed using Seqman software (DNAstar, Madison, WI).

## Results

A total of 33 women with TNBC were included in the study. The average age at diagnosis was 53.8 (range 33–82 years). Among the selected cases, nine (27.3%) had a family history for cancer: eight had a family history of breast cancer and one women had a family history of prostate cancer. The remaining 24 patients (72.7%) were sporadic cases (Table 1). The 5382inC mutation was identified in two out of 33 women with TNBC, representing an overall prevalence of 6% (2/33). The two 5382inC mutation carriers had a family history of breast cancer. Thus, the detection rate of the 5382inC mutation among TNBC women with family history of breast cancer was 25% (2/8). The two 5382inC mutation carriers were postmenopausal and diagnosed at the age of 50 and 57. When stratified by age, the frequency of BRCA1 mutation was 8.7% (2/23) in patients diagnosed at age ≥ 50 years and 0% in those diagnosed < 50. The characteristics and family history of the 5382inC mutation carriers is shown in Table 2.

**Table 2:** Characteristics and family history of the 5382inC mutation carriers

Patient ID	Age at diagnosis	Menopausal status	SBR grade	Lymph node status	Family history of breast cancer
P1	50	Menopausal	3	N+	Mo Br, S Br, MA Br
P2	57	Menopausal	2	N−	S Br

*Br* Breast cancer, *MA* Maternal Aunt, *Mo* Mother, *S* Sister

## Discussion

In the present study, the recurrent 5382inC BRCA1 germline mutation was identified in two postmenopausal women with TNBC diagnosed at the age of 50 and 57 and presenting a family history of breast cancer. Overall, the frequency of the 5382inC mutation in TNBC patients was 6% (2/33). The detection rate of the 5382inC mutation among TNBC women with family history of breast cancer was 25% (2/8).

Data from a previous Tunisian study showed that five out seven (71%) breast cancer patients with deleterious BRCA1 mutation exhibited triple negative tumors. Interestingly, all the three cases carrying the 5382insC mutation display a triple negative phenotype [11]. Taken together, these data confirm a noticeable contribution of the BRCA1 5382insC recurrent mutation in TNBC development in Tunisia. Our data indicate that screening for 5382insC mutation in the *BRCA1* gene may be of interest in genetic testing in our population. Such an approach will allow for a more rapid and less expensive test and would help to identify Tunisian women at high-risk of developing TNBC.

In Tunisia, the *BRCA *genetic testing is still costly and not broadly available. Thus more effort should be done to select which individuals should be offered *BRCA* testing. Currently, in our population, the selection criteria of familial cases are primarily based on age at diagnosis and the number of affected first- and second-degree relatives with breast or ovarian cancer.

In our study, cases were selected based on triple negative status and not on age at onset or family history. Using only the receptor triple negativity as a selection criterion allowed us to identify 25% (2/8) of BRCA1 mutation carriers with a family history of breast cancer. In addition, one of the two 5382insC mutation carriers was a postmenopausal woman diagnosed with TNBC at the age of 57 and having only one first-degree relative with breast cancer. According to current selection criteria in Tunisia, this woman may not be eligible for *BRCA* testing. Thus, the present findings highlight that adding receptor triple negativity to the traditional risk factors of age and family history could help to identify BRCA1 mutation carriers, mainly those with a masked family history.

Some Tunisian studies aiming to define predictive factors for BRCA mutations were undertaken [7, 13]. In line with our data, Riahi et al. indicated that besides family history, diagnosis before the age of 40, triple negative subtype and age at menarche could be the effective selection criteria for *BRCA *genetic test in our population [7].

In agreement with our study, Zhang et al. suggested that Chinese women with familial breast cancer whose tumors were diagnosed with triple-negative phenotype were good candidates for *BRCA1/2 *testing [14]. Moreover, several studies have shown that applying current guidelines for genetic testing may lead to overlook some proportion of BRCA mutation carriers and suggest that besides a positive family history of cancer or early age of diagnosis, receptor triple negativity should be considered in the guidelines for genetic analysis of *BRCA1* and *BRCA2 *[15–18].

Given the strong association between TN phenotype and BRCA1 mutations, several studies have evaluated the prevalence of BRCA1 mutations in patients with TNBC. Reports indicate that the prevalence of BRCA1 mutations in TNBC patients varies between populations and from one study to another. Most studies included patient populations selected for young age at diagnosis (under the age of 50 or 40 years) and reported a prevalence ranging from 7.6 to 23% [19–22]. Other studies evaluated the frequency of BRCA1 mutations in unselected TNBC patient populations. Hartman et al. evaluated a cohort of 199 unselected women with TNBC, the BRCA1 mutation frequency was 6.5%. When stratified by age, the frequency of BRCA1 mutations was 2.8% in patients diagnosed at age ≥ 50 years [16]. The study conducted by Rummel et al. showed that BRCA1 mutations were detected in 9% of unselected TNBC patients and in 4.8% of women diagnosed at age ≥ 50 years [23]. In the largest study to date of unselected TNBC patients (n = 1824), 8.5% had a mutation in *BRCA1*. For the 50–59 age group, the prevalence was 7.4% [18]. In the present study, 6% (2/33) of our TNBC patients unselected for age and family history were found to carry the 5382insC mutation. The mutation frequency was 8.7% (2/23) in women diagnosed at age ≥ 50 years. Although we screened only one BRCA1 mutation in a relatively small cohort, the overall mutation frequency (6%; 2/33) is similar to the study by Hartman et al., [16]. However, compared to the above studies, the mutation frequency was higher in our group of women diagnosed at age ≥ 50 years (8.7%; 2/23). This may be partly explained by the high proportion of women with family history of breast cancer in this group (4/23, 17%).

The two main limitations in the present study are the small sample size and the screening of a single BRCA1 mutation (5382insC). Thus, our data underscore the need for larger series and the full screening of the *BRCA1* gene to better understand the contribution and the frequency of BRCA1 mutations in individuals with TNBC.

TNBC patients with BRCA1 mutations require different strategies for patient management, counseling, and treatment. Thus, improved understanding of the frequency of BRCA1 mutations in patients with TNBC will impact their clinical management.

## Conclusions

The present data confirm a noticeable contribution of BRCA1 5382inC mutation in TNBC development in Tunisia and further indicate that screening for 5382insC mutation in the *BRCA1* gene is of great interest in genetic testing in our population. Our data highlight that receptor triple negativity could be an effective selection criterion for *BRCA* genetic test in our population and should therefore be considered in genetic testing guidelines in Tunisia.

## Abbreviations

BC: breast cancer
TNBC: triple negative breast cancer
BRCA1: breast cancer 1
BRCA2: breast cancer 2
HER2: human epidermal growth factor receptor-2
IARC: International Agency for Research on Cancer
ER: estrogen receptor
PR: progesterone receptor
PCR: polymerase chain reaction
